# Using Technology to Enhance Teaching and Learning in Pharmacy Education

**DOI:** 10.3390/pharmacy9030150

**Published:** 2021-09-03

**Authors:** Clark D. Kebodeaux, Vivienne Mak

**Affiliations:** 1 College of Pharmacy, University of Kentucky, Lexington, KY 40508, USA; 2 Faculty of Pharmacy and Pharmaceutical Science, Monash University, Parkville 3052, Australia

It was a privilege to serve as guest editors in Pharmacy for the Special Issue ‘Technology-Enhanced Pharmacy Teaching and Learning Strategies’. The outbreak of the SARS-CoV-2 virus and resulting COVID-19 pandemic has taken a unique toll on the global community. In addition to the threat the virus presented to overall public health, countries and communities were forced to adapt to a new paradigm in an attempt to reduce the spread of the SARS-CoV-2 virus. These mitigation strategies required significant and sudden change, particularly in education. While online-based learning is not entirely foreign to students prior to the pandemic, teachers and learners were forced to adapt existing pedagogical approaches to facilitate learning without physical proximity. This was challenging for health profession schools and colleges around the world with experiential education being particularly impacted. Given the unique responsibility healthcare professions have to help to overcome the pandemic, it was critical that these institutions continue their mission while reducing risk of infection and helping patients obtain necessary treatment, testing, and vaccination against novel infection disease.

This special issue highlights innovative practices within schools and colleges of pharmacy-on-pharmacy education—specifically during the COVID-19 pandemic. While there have been many perspectives published on the use of technology in education during and after the pandemic [1,2], this section highlights many innovative practices that educators and practitioners alike may benefit from long after the COVID-19 pandemic.

An excellent example of a pedagogical approach that will continue long after the pandemic is innovative ways to help students understand Metacognition. Authors used ‘concept mapping’ in order to help students effectively understand disease processes and the associated treatments. This study focused on a qualitative analysis of a software program to validate concept maps. Featuring analysis of chronic kidney disease (CKD), rheumatoid arthritis (RA), heart failure, and asthma; faculty were able to assess the number of nodes and links (connections in the maps) against expert keys as a way of automating assessment for a complex learning activity performed by students as part of active learning [3].

The most common topic addressed in the special issue was the use of simulation, particularly around recreating authentic, simulation-based experiences. Simulation allowed student pharmacists to experience the medication dispensing process especially when they were not allowed to complete placements, or clinical experiences, within needed practice sites due to COVID-19 precautions. The most common software used was ‘MyDispense’, developed by Monash University in Melbourne, Australia, and used around the world to teach this medication use process [4,5]. One article focused on the impact of MyDispense in introductory experiential education [6]. Using a randomized comparison of students with various levels of experience in community-based settings, students who utilized simulation received higher marks from their preceptors—specifically for self-care and medication counseling. Another detailed analysis of MyDispense focused on the errors made by introductory students in simulating the dispensing process [7]. Students were more likely to make errors related to prescription labeling than interpreting numbers (medication quantity, repeats) and dispensing errors were classified by harm including route of administration as well as frequency of dosing. Simulation was found to be an important tool to allow these students to learn from mistakes and avoid preventable errors in practice. Lastly, this tool was used to teach specific instructions related to pharmacy law [8]. Students who used this simulation noted a better understanding of the law as well as more interest in course materials. Specifically, the findings of this study led to increased utilization throughout the curriculum to reinforce understanding of pharmacy law.

Pharmacy educators have an extensive history of innovation in assessments including competency-based assessments [9]. In order to meet the demands of the practice environment, educators employ a variety of assessment techniques that focus on patient care and simulation to replicate practice environments. However, continuing simulated assessments posed a significant challenge during the COVID-19 pandemic. Many schools and colleges of pharmacy employ the use of Objective Structured Clinical Examinations (OSCEs) to assess competency. Due to the COVID-19 challenges, educators found innovative ways to design virtual assessments to measure the same level of student performance. For example, a three-station OSCE was performed in a first-year student cohort where faculty specifically sought the feedback from student pharmacists after implementing remote OSCEs [10]. Responses were collected after the assessment with a significant response rate and authors coded for thematic analysis. Key findings were categorized by domains of ‘learned’, ‘liked’, and ‘suggest’—the most prominent themes across the categories were logistics, followed closely by technology and skill development. Faculty implementing remote OSCEs needed to place additional effort and focus on logistics when in the remote environment. A case report published in the special issue also reinforced the need to take a scholarly approach to teaching and learning. Effective online ‘presence’ including module design, effective dialogue and replicating human interaction were crucial teaching strategies in the development of Professionalism-and-Ethics courses in a remote environment [11].

Two studies focused on the impact of online assessments and outcomes as a result of the pandemic. An excellent example of remote assessments during the pandemic compared the outcomes of a communication assessment from 2019 to 2020 as well as an OSCE conducted during the first year of the pharmacy curriculum [12]. Faculty were able to provide a comparison that revealed students scored higher in the virtual format for a communication assessment (overall and individual variables) but only in one variable for the remote OSCE (establishing trust). All other variables of the OSCE, including empathy, professionalism, and total score were unchanged from in-person to remote assessment. A similar analysis was performed within the same semester of a simulation laboratory for first- and second-year student pharmacists [13]. In order to accommodate the simulation format of the clinical laboratory course, students were divided into tracks that completed a 4-week module at separate times during the semester. As a result of the pandemic, tracks that completed in-person vs. online assessments showed no differences in outcomes measured in terms of required remediation. More research should be performed in this area to continue to evaluate various methods of assessment in pharmacy education.

Technology was also used to replicate experiential learning for international students in pharmacy education [14]. Students were required to complete a 2.5 credit-hour course in order to meet their introductory pharmacy practice experiences (IPPEs) requirements in the Summer of 2020. A variety of tools were used including a dedicated Learning Management System (LMS), video technology, as well as assignments and activities related to electronic health records (EHR) and dispensing (MyDispense). Students had to complete over 200 h remaining to meet United States Accreditation for Pharmacy Education (ACPE) standards for introductory experiences. Overall, students’ perceptions of the experience were high, including organization and expectations; however, students still had a preference for live learning compared to simulation if possible.

While previous articles focused on individual coursework, assessment, or innovative teaching approaches, there are important lessons to be learned about the role of technology on the impact on the overall curriculum, including clinical experiences. Faculty performed an analysis of strategic plans for accredited schools and colleges of pharmacies in both Canada and the United States to identify the inclusion of technology priorities within published plans. Given how technology became more important for all institutions during the COVID-19 pandemic, authors found that just over half of identified strategic plans (53.2%) had a goal or objective related to technology. Other innovations used by authors in this Special Issue include the use of dashboards within various competencies in pharmacy education [15]. Dashboarding is not common in education; however, it can be a potentially beneficial approach to utilize technology, feedback, and data to analyze course performance as well as areas of needed intervention. For example, group outcomes can be tracked, including time to achieving competency, class comparisons, and the ability to use continuous feedback for improvement. This is an excellent example of methodology that will continue to be useful long after the COVID-19 pandemic.

Technology-enhanced pharmacy teaching and learning strategies were practiced long before the COVID-19 pandemic; however, the drastic change that occurred due to the pandemic may serve as a catalyst for implementation of new technologies. Continued evaluation and sharing of best practices, educational outcomes, and innovative strategies are paramount to implementing new strategies in effective ways. The articles in this Special Issue exemplify great examples of innovations in teaching and learning utilizing technology and we look forward to continuing these efforts in the future.

## Data Availability

Not applicable.
